# Frailty Screening and Prehabilitation Before Kidney Transplant Listing: A Practical Narrative Review

**DOI:** 10.7759/cureus.101597

**Published:** 2026-01-15

**Authors:** Fahad S Alrashidi, Muthbat A AlDawsari, Helmi A Negm, AbdulAziz A AlAtmi

**Affiliations:** 1 Department of Internal Medicine, Nephrology Section, Al-Diriyah Hospital, Riyadh Third Health Cluster, Riyadh, SAU

**Keywords:** cancer functional assessment set, clinical frailty, improved ckd stage, • kidney transplantation, transplant candidacy

## Abstract

Frailty is increasingly recognized as a clinically meaningful and potentially modifiable vulnerability among kidney transplant candidates and among patients with advanced chronic kidney disease (CKD) referred for transplant evaluation. Contemporary guidance emphasizes individualized risk-benefit assessment and equity in access to transplantation while acknowledging substantial inter-center variability in evaluation practices. Frailty assessment may improve transparency and consistency compared with subjective impressions, support shared decision-making, and identify targets for prehabilitation and other optimization strategies. However, challenges persist, including heterogeneity in frailty definitions, variability in reported frailty prevalence across transplant-candidate cohorts and instruments, uncertainty regarding optimal tools and thresholds, and operational questions regarding who should measure frailty, when to repeat testing, and how to interpret results without creating unjust barriers to listing. This narrative review summarizes (i) conceptual models of frailty relevant to transplant candidates, (ii) practical frailty assessment options and their feasibility in transplant clinics, (iii) a pragmatic workflow for integrating frailty screening into pre-listing evaluation, and (iv) evidence and implementation considerations for prehabilitation in kidney transplant candidates. Overall, frailty screening is best positioned as a tool for decision-support and targeted optimization rather than a rigid exclusion criterion.

## Introduction and background

Evaluation of candidates for kidney transplantation is increasingly understood as a multidimensional process that extends beyond identifying comorbid conditions. Contemporary assessments must also address functional capacity, cognitive performance, psychosocial readiness, adherence potential, and the anticipated net benefit of transplantation compared with continued dialysis or conservative management. This broader perspective is essential because key determinants of post-transplant outcomes, such as perioperative resilience, capacity to manage complex post-operative care, and recovery trajectory, are only partially captured by traditional risk markers, including age, diabetes, or cardiovascular disease [1,2].

Frailty, broadly defined as diminished physiologic reserve and increased vulnerability to stressors, has emerged as a clinically meaningful construct that may better reflect “biologic age” and surgical resilience than chronological age alone. Frailty is distinct from disability and comorbidity; it represents a vulnerability state arising from multisystem dysregulation and often manifests as weakness, slowness, low activity, exhaustion, and unintentional weight loss or sarcopenia [3-5]. In chronic kidney disease (CKD) and dialysis cohorts, frailty is common, tends to manifest earlier than in the general population, and is consistently associated with adverse outcomes such as hospitalization, falls, reduced quality of life, and mortality. These associations are biologically plausible in advanced CKD, given inflammatory and metabolic disturbances, protein-energy wasting, anemia, neurocognitive effects of uremia, and accelerated muscle loss [5]. Importantly, transplant-candidate cohorts also demonstrate a substantial burden of frailty, with reported prevalence varying by instrument, timing of assessment, and case-mix (e.g., phenotype-based vs deficit-accumulation or brief screening tools) [6]. Because tool choice materially influences who is labeled as frail or prefrail, frailty findings should be interpreted in the context of the underlying construct being measured, rather than being treated as interchangeable across instruments [6].

Within kidney transplantation, frailty assessment has been increasingly advocated to enhance objectivity and transparency in candidate evaluation, improve risk communication, and identify potentially modifiable impairments suitable for targeted intervention. Structured assessments can complement clinical judgment by standardizing recognition and documentation of functional vulnerability, thereby reducing reliance on subjective impressions that may vary by provider, setting, or patient advocacy [4,6]. In transplant candidates, frailty status has been associated with outcomes relevant to patients and programs, including early postoperative complications, prolonged hospitalization, delayed recovery, rehospitalization, and challenges with post-transplant care demands, although much of the current evidence base remains observational, and definitive interventional outcome data remain limited [4-6]. Accordingly, frailty metrics are best used to support individualized counseling regarding anticipated recovery, caregiver needs, and the potential trade-offs between immediate transplantation and a period of optimization [4,6].

Despite growing interest, legitimate barriers have slowed routine implementation. Frailty measures vary widely in conceptual model, required equipment, staff training, and time burden; thresholds for “high risk” are not uniform; and busy clinic workflows may not accommodate complex performance testing [4,6]. Programs also recognize the ethical risk of inadvertently restricting access, particularly for older adults, women, socioeconomically disadvantaged patients, and those with disabilities, if frailty is used as a simplistic exclusion criterion rather than as one component of holistic evaluation [1,2,4,6]. These concerns underscore the need for an implementable approach that is feasible, reproducible, and explicitly focused on risk stratification and optimization, not automatic gatekeeping.

Therefore, frailty screening in kidney transplant evaluation should be positioned as a clinical decision-support tool that clarifies perioperative and post-transplant risk, identifies domains for intervention (e.g., strength, nutrition, mobility, cognition, and psychosocial support), and triggers targeted prehabilitation pathways to enhance readiness for surgery and postoperative recovery [4,6]. This approach aligns with the overarching goals of transplant medicine: maximizing benefit while upholding equity, transparency, and patient-centered care [1,2].

This review provides a pragmatic synthesis for integrating frailty screening into pre-listing kidney transplant evaluation and for linking results to structured optimization pathways. Major frailty constructs and measurement options relevant to transplant clinics are summarized, workflow models are outlined, and implementation strategies are proposed for operationalizing frailty findings into interdisciplinary interventions. Emphasis is placed on safeguards to minimize inequity and ensure that frailty assessment enhances, rather than restricts, access to transplantation [1,2,4,6].

Methods

We performed a pragmatic narrative evidence scan using PubMed/MEDLINE (Medical Literature Analysis and Retrieval System Online) and Google Scholar (approximately 2008-2025) with keywords related to frailty measurement, kidney transplant candidate evaluation, and transplant prehabilitation. PubMed/MEDLINE was used to identify peer-reviewed transplant and nephrology studies, while Google Scholar was used as a supplementary tool to capture (i) society statements, commentaries, and implementation literature that may be inconsistently indexed in MEDLINE and (ii) citation chaining(backward reference screening and forward citation tracking) from seminal transplant-frailty and prehabilitation papers. No protocol was registered, and no formal risk-of-bias assessment was performed, consistent with a narrative review. Because this was a narrative rather than a systematic review, Preferred Reporting Items for Systematic Reviews and Meta-Analyses (PRISMA) reporting and a study selection flow diagram were not used.

We prioritized four evidence streams: (i) kidney transplant candidate evaluation guidance and transplant program commentary relevant to functional and geriatric assessment; (ii) major frailty conceptual frameworks and the frailty instruments most commonly operationalized in CKD and transplant candidate populations; (iii) evidence syntheses and primary studies describing associations between frailty measurement and clinically relevant outcomes in CKD, dialysis, and transplant candidate/recipient cohorts; and (iv) interventional studies and reviews evaluating prehabilitation or optimization strategies in kidney transplant candidates and closely related surgical populations [7-10]. For each tool and framework, we abstracted domains captured (physical performance, function, cognition, symptom burden), administration requirements, typical completion time, and potential sources of measurement bias (e.g., language, education, baseline disability), and we mapped how results could plausibly trigger structured optimization pathways (e.g., physical therapy (PT)/occupational therapy (OT) referral, nutrition optimization, medication review, psychosocial support). When evidence was heterogeneous or conflicting, we emphasized convergent signals, reported trade-offs explicitly, and avoided implying that any single instrument is universally “best” across programs.

## Review

Frailty: definitions and conceptual models relevant to transplantation

Two dominant paradigms have shaped clinical frailty assessment and are particularly relevant to transplantation because they operationalize vulnerability in different and potentially complementary ways. The phenotypic (Fried) model defines frailty as a syndrome characterized by measurable physical features (typically including weakness, slowness, exhaustion, low activity, and unintentional weight loss). Its strengths for transplant evaluation include relative objectivity and strong face validity for perioperative resilience, where physical reserve and recovery capacity are central [3,11]. A practical advantage is the distinction between pre-frailty and frailty, enabling earlier intervention and longitudinal monitoring aligned with prehabilitation workflows.

By contrast, the deficit accumulation model conceptualizes frailty as the cumulative burden of health deficits across multiple domains (symptoms, comorbidities, functional limitations, cognition, and psychosocial factors). This model is commonly operationalized through frailty indices; a widely used clinical derivative is the Clinical Frailty Scale (CFS), which provides a rapid, clinician-anchored estimate of baseline fitness and dependency [7,9]. Deficit-based approaches often align naturally with global risk stratification because they integrate multi-domain vulnerability; however, they may be more susceptible to inter-rater variability and documentation quality, and they can conflate frailty with stable disability if baseline function and supports are not clearly contextualized.

Because these paradigms capture different constructs, the same transplant candidate may be classified differently across instruments. Rather than treating discordance as error, discordant results should be interpreted as signaling domain-specific vulnerability (e.g., impaired physical reserve vs broader multidomain complexity). In practice, a feasible approach is sequential use: a brief global screen (e.g., CFS or a short questionnaire-based tool) to triage candidates for additional assessment, followed by an objective performance-anchored measure when screening is positive, borderline, or discordant with clinical impression. This two-tier strategy can improve interpretability while minimizing clinic burden and helps ensure that frailty findings trigger a standardized optimization response plan rather than functioning as a de facto exclusion criterion.

Why frailty manifests early in CKD and why it matters for transplant evaluation

In CKD, frailty is not simply a reflection of chronological age. Uremia and chronic inflammation, protein-energy wasting and sarcopenia, anemia and reduced exercise tolerance, metabolic derangements (including acidosis and mineral-bone disease), and high cardiovascular burden interact to erode physiologic reserve and amplify vulnerability to stressors. Dialysis-related factors may further contribute through intermittent volume shifts, intradialytic symptoms, fatigue, sleep disruption, and reduced opportunity for sustained conditioning, while multimorbidity and polypharmacy can exacerbate falls risk, delirium susceptibility, and functional decline [12-16]. Although much of this mechanistic and epidemiologic evidence is derived from CKD/dialysis cohorts, it provides a biologic rationale for the frailty observed in transplant candidate populations and underscores why vulnerability may be clinically meaningful even in younger adults with advanced kidney disease.

From a transplant perspective, frailty is clinically consequential because it plausibly intersects with several high-impact outcomes: perioperative complications, prolonged length of stay, delayed functional recovery, rehospitalization, and the ability to sustain the demanding post-transplant regimen (medications, monitoring, infection-prevention behaviors, and frequent follow-up) in the early vulnerable months after transplantation. The practical question for transplant teams is therefore not whether frailty exists, but how to measure it efficiently and respond in ways that improve readiness and recovery while preserving equitable access.

Practical frailty assessment tools in transplant clinics

A recurring barrier to routine frailty screening is uncertainty about which tool is most appropriate for kidney transplant candidates. The current evidence base supports multiple approaches, each with predictable trade-offs in time, training, objectivity, and domain coverage. For pragmatic implementation, tools can be grouped into four clinic-relevant categories: (i) Performance-based measures (e.g., gait speed, chair stands, Short Physical Performance Battery (SPPB)) provide objective, reproducible markers of physical reserve and are relatively sensitive to change, making them useful for baseline risk stratification and for monitoring response to optimization pathways; (ii) Phenotype-based instruments (e.g., Fried components) combine performance and symptom/activity measures and are widely used in transplant research, but may be more time-intensive depending on how components are operationalized; (iii) Clinician-anchored global scales (e.g., CFS) are rapid and low-burden, but require clear anchoring to baseline function and consistent staff calibration to minimize inter-rater variability and avoid conflating disability with frailty; (iv) Deficit-based indices and multidomain screens can capture broader vulnerability (including cognition and function) but may depend on robust data capture and can be less feasible in short, high-throughput clinics.

Because no single instrument is optimal across all programs, a practical selection framework is to match the tool to clinic constraints and intended use: brief screening for all candidates, confirmation/characterization in those who screen positive or have discordant findings, and standardized referral pathways tied to modifiable domains (strength, mobility, nutrition, symptom control, cognition, psychosocial supports). In many settings, a two-tier workflow is most implementable: a rapid global or single-domain screen embedded into routine vitals/rooming, followed by a short objective performance test (or phenotype assessment) in those with concerning results. While improvement in performance metrics and frailty scores is a meaningful intermediate target and supports readiness-focused care, evidence that score changes causally translate into long-term graft or survival benefits remains evolving; therefore, frailty measurement is best operationalized to trigger optimization and shared decision-making rather than rigid listing thresholds.

Table 1 summarizes commonly used instruments that can be integrated into transplant evaluation with minimal disruption, highlighting administration time, required equipment, staff role, key domains captured, and how results can trigger prehabilitation pathways.

**Table 1:** Practical frailty assessment tools applicable to kidney transplant candidates PFP, Physical Frailty Phenotype; CFS, Clinical Frailty Scale; SPPB, Short Physical Performance Battery; FI, Frailty Index; FRAIL, Fatigue, Resistance, Ambulation, Illness, Loss of weight; CKD, chronic kidney disease; EHR, electronic health record; PT/OT, physical therapy/occupational therapy; QI, quality improvement

Instrument (reference)	What it captures	Practical requirements	Key strengths	Key limitations	Suggested use in transplant evaluation
Fried Physical Frailty Phenotype (PFP) [3]	Physical frailty phenotype	Moderate; grip strength + walking test + questionnaire	Widely studied; intervention-targeted (strength, activity)	More time; requires measurements; may be affected by CKD symptoms	Baseline objective assessment when resources allow; research-ready
Clinical Frailty Scale (CFS) [7]	Deficit accumulation (global clinical judgment anchored to function)	Very fast; no equipment	High feasibility; captures function/cognition broadly	Inter-rater variability; training needed; less granular	First-line screening in busy clinics; triggers further objective tests
FRAIL scale [10]	Simple screening (fatigue, resistance, ambulation, illness, weight loss)	Very fast; no equipment	Extremely feasible; good for rapid triage	Screening only; less precise	Initial screen; use with objective follow-up if positive
Short Physical Performance Battery (SPPB) [8]	Lower extremity performance (balance, gait speed, chair stands)	~7–10 minutes; chair + stopwatch	Objective; sensitive to functional change	Requires space/time; needs standardized administration	Second-line objective test; useful for monitoring response to rehab
Grip strength (component of PFP) [3]	Muscle strength surrogate	Fast; dynamometer	Objective; predictive in many cohorts	Requires device; thresholds vary	Add-on measure if full PFP not feasible
Deficit-based Frailty Index (FI) (conceptual) [9]	Accumulation of deficits across domains	Variable; data-driven	Broad domain capture	Requires data infrastructure; heterogeneity	Research/QI when EHR-based automation is possible

When and how to use frailty results in listing decisions

A balanced approach is to incorporate frailty into evaluation as part of whole-patient assessment while avoiding simplistic thresholds that become de facto exclusion criteria. The kidney transplant listing debate literature emphasizes both the rationale for measuring frailty (prevalence, prognostic value, and reduction of subjective bias) and the counterarguments (lack of standardization and uncertainty in operationalizing results) [4].

**Figure 1:**
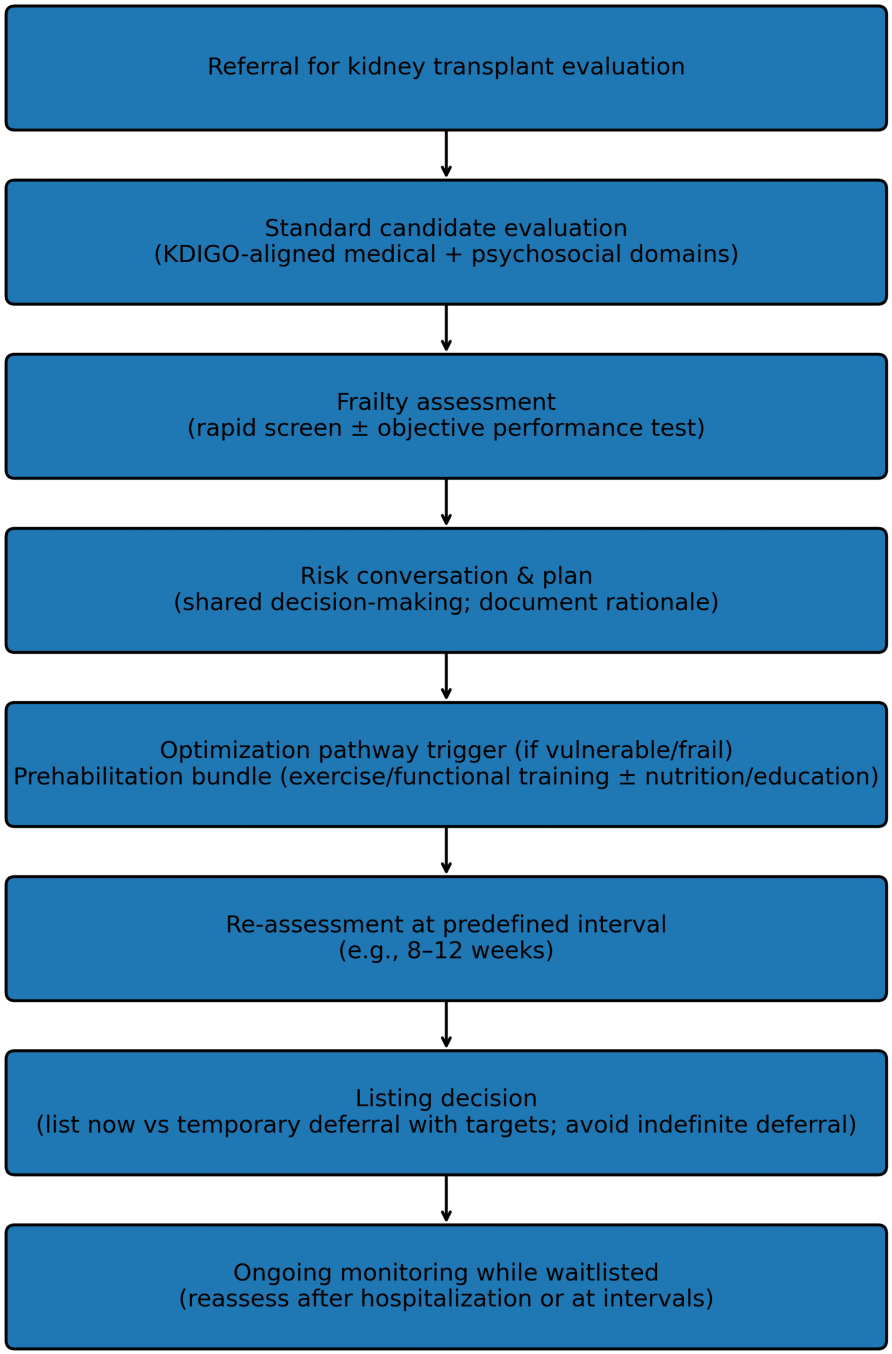
Frailty pre-listing workflow for kidney transplant candidates. KDIGO, Kidney Disease: Improving Global Outcomes

A practical workflow is shown in Figure 1, emphasizing standardized screening, multidisciplinary interpretation, and targeted optimization rather than automatic delisting.

Prehabilitation in kidney transplant candidates: rationale and evidence

Prehabilitation aims to improve physiologic reserve before an anticipated stressor (e.g., major surgery or transplantation) through structured, time-limited interventions-most commonly exercise training, nutrition optimization, and functional support. Conceptually, it aligns tightly with frailty paradigms because many frailty domains are at least partially modifiable, including muscle strength and power, gait speed and balance, aerobic capacity, nutrition status, symptom burden (fatigue, sleep disturbance), and functional confidence. In kidney transplant candidates, prehabilitation is particularly attractive because candidates often exhibit potentially reversible contributors to vulnerability (e.g., anemia, metabolic acidosis, protein-energy wasting, dialysis inadequacy, volume instability, deconditioning, depression/anxiety, and polypharmacy). These factors can compound, producing a cycle of inactivity and functional decline that may worsen over time on the waitlist.

The clinical rationale is twofold. First, prehabilitation may reduce perioperative risk by improving cardiorespiratory fitness, strength, and functional reserve-mechanisms with face validity for better tolerance of surgery, fewer complications, and faster recovery. Second, it may improve “transplant readiness,” including the ability to attend visits, maintain adherence, mobilize early after surgery, and engage in post-transplant self-management. Importantly, prehabilitation reframes frailty screening from a passive risk label to an active management pathway: identifying vulnerability becomes the starting point for optimization rather than a justification for exclusion.

However, transplantation introduces a unique operational challenge compared with elective surgery: timing is uncertain, and candidates may remain on the waitlist for months to years with intermittent illness, dialysis access events, hospitalizations, and shifting social circumstances. This volatility complicates traditional center-based rehabilitation models, which typically rely on predictable surgical scheduling and sustained attendance.

Early transplant-focused discussions framed prehabilitation as a potential lever to expand access and improve outcomes, while emphasizing the limitations of the evidence base and the practical difficulty of delivering structured programs to a waitlisted population with fluctuating timelines and competing burdens of care [13]. Subsequent pilot work supports feasibility and has demonstrated improvements in intermediate endpoints such as physical activity and objective function measures (e.g., walk performance and/or composite physical performance metrics) after structured prehabilitation delivered before transplantation [14]. More recently, multicenter studies have evaluated home-based or hybrid models, which may be particularly scalable for candidates facing transportation constraints, work/family obligations, or limited access to supervised rehabilitation services [15]. Collectively, the emerging evidence supports feasibility and signals potential benefit, but it remains largely composed of observational analyses, small pilots, and implementation studies. Accordingly, while improvements in functional performance or frailty scores are clinically meaningful intermediate targets and may plausibly translate into improved perioperative tolerance, definitive evidence that prehabilitation-driven changes in frailty metrics causally improve “hard” outcomes (e.g., perioperative complications, graft outcomes, or survival) is still evolving [13-16]. Larger trials are therefore needed to clarify optimal intensity and duration, identify which subgroups derive the greatest benefit (e.g., frail vs pre-frail; older vs younger; dialysis vs pre-dialysis), define meaningful endpoints (waitlist events, transplant access, perioperative complications, length of stay, and patient-reported outcomes), and establish cost-effectiveness and implementation sustainability [16].

From an implementation standpoint, a pragmatic interpretation is that prehabilitation is best positioned as a structured, multidisciplinary optimization pathway for vulnerable candidates, especially those with modifiable deficits, rather than a one-size-fits-all program. Tiered models (low-, moderate-, and high-intensity pathways) that match intervention intensity to frailty severity, symptom burden, and social feasibility are likely to be most implementable in routine practice. Embedding standardized reassessment intervals and explicit referral triggers can also help ensure that frailty measurement functions as a pathway to optimization and shared decision-making, not as a rigid listing threshold.

Implementation considerations: staffing, workflow, and equity

Several implementation principles can improve uptake and reduce unintended harms. First, programs should standardize measurement and documentation, including when the tool is administered (pre-listing vs listing vs annual review), who administers it, and how results are recorded, so that frailty data are reproducible and interpretable across providers and over time. Second, programs should interpret results in a clinical context, explicitly accounting for temporary illness, recent hospitalization, dialysis vintage, language barriers, baseline disability, and social supports, to avoid misclassification and inappropriate conclusions.

Third, screening should always trigger an actionable pathway. This is the operational distinction between frailty as a risk label and frailty as a management construct. Action pathways can include: nutrition assessment and supplementation, physiotherapy/occupational therapy referral, individualized exercise prescription, anemia and metabolic optimization (e.g., acidosis, mineral-bone parameters), dialysis adequacy and symptom review, medication reconciliation with deprescribing where appropriate, and structured falls-risk mitigation. Clear referral thresholds and standardized order sets materially improve throughput and reduce clinician hesitation in adopting screening.

Equity must be explicitly engineered into the workflow. Frailty screening should be designed to protect equity rather than restrict access, with guardrails that prevent scores from functioning as rigid exclusion thresholds, particularly for older adults, women, socioeconomically disadvantaged candidates, and those with limited transportation or caregiver resources [4,15]. Home-based or tele-supported prehabilitation models can reduce access barriers and may mitigate differential drop-out that can occur with center-based programs. Finally, multidisciplinary ownership (transplant nephrology, surgery, nursing coordination, dietetics, physiotherapy, and social work) improves feasibility, distributes workload, and ensures that identified deficits are matched to services the program can actually deliver.

Discussion

Frailty measurement is increasingly aligned with the direction of kidney transplant candidate evaluation: toward individualized, transparent, multidomain assessment that better reflects biologic reserve and recovery capacity than comorbidities or chronological age alone [1,2]. A central rationale for incorporating frailty is its potential to replace inconsistent subjective impressions with measurable constructs, improve risk communication, and identify modifiable targets for optimization [4-6]. However, the value of frailty depends on how it is implemented. If programs use frailty scores as rigid exclusion thresholds, screening can become an equity threat rather than an equity tool, potentially amplifying disparities rather than improving outcomes [4]. Accordingly, frailty should be positioned as decision-supportthat prompts structured optimization and shared decision-making, not automatic gatekeeping.

A pragmatic implementation model is feasible screening with selective objective testing (Table 1), embedding results into multidisciplinary review, and linking positive or concerning findings to time-bound optimization and prehabilitation pathways (Figure 1). Tool choice should be driven by (i) the construct of vulnerability a program intends to capture (physical reserve vs multidomain complexity), (ii) operational feasibility (time, equipment, staffing, workflow), and (iii) the program’s capacity to respond with actionable interventions. In other words, measurement should be paired with the ability to act.

An important practical issue is that phenotype-based and deficit-accumulation frameworks capture overlapping but distinct vulnerabilities and may classify the same patient differently. Discordance should be interpreted scientifically as a domain-specific signal, not necessarily a measurement failure: phenotype or performance measures may preferentially reflect physical reserve and recovery potential, whereas deficit-based measures may better contextualize multidomain complexity, dependency, and care needs. When results are discordant or borderline, particularly when there is baseline disability, language/education limitations, or variable documentation, programs should prioritize interpretability by anchoring assessments to baseline function and supports and by using objective performance measures when feasible [8]. A two-tier strategy is often the most implementable: a rapid screen embedded into routine workflow (e.g., rooming/vitals) followed by a short objective performance assessment (e.g., gait speed, chair stands, SPPB, or phenotype components) for candidates who screen positive, have borderline scores, or have discordant clinical impressions. This sequential approach reduces burden, improves transparency, and supports standardized downstream referral.

Evidence on formal “combined” frailty modeling in transplant candidates remains limited, and existing studies show that different instruments can have modest agreement and capture different risk dimensions [8]. Rather than asserting that any combination is universally superior, a defensible approach is to combine measures operationally (screen → characterize → intervene) and ensure that each step maps to an actionable response plan (PT/OT, nutrition, symptom optimization, psychosocial support, caregiver planning). This preserves feasibility while improving interpretability and aligns with equity safeguards by avoiding single-score thresholds used in isolation.

The evidence base for prehabilitation in kidney transplant candidates is growing, including pilot and multicenter work supporting feasibility and improvements in intermediate endpoints, particularly with home-based or hybrid approaches that may mitigate transportation and scheduling barriers [17-20]. At present, most transplant-specific prehabilitation evidence remains observational or pilot-scale, and key questions remain regarding which candidates benefit most, how to sustain engagement during prolonged waitlisting, what intensity and duration are required, and which outcomes should be prioritized (waitlist events, transplant access, postoperative complications, length of stay, graft outcomes, and patient-reported outcomes). Importantly, while improvement in functional performance or frailty metrics is a clinically meaningful intermediate target and plausibly linked to perioperative tolerance, definitive evidence that prehabilitation-driven changes in frailty scores causally translate into improved graft or survival outcomes is still evolving [17-20]. Until larger trials mature, implementing prehabilitation as a structured quality-improvement pathway for vulnerable candidates-explicitly oriented toward optimization, shared decision-making, and equitable access-remains defensible and clinically meaningful.

Limitations

As a narrative review, this article does not provide a graded appraisal of evidence; no formal risk-of-bias assessment was performed, and the synthesis should be interpreted as pragmatic implementation considerations rather than definitive practice guidelines. The included literature may be subject to selection bias and publication bias, and heterogeneity across studies (frailty definitions, instruments, thresholds, timing of assessment, and outcomes) limits direct comparability and precludes quantitative pooling. In addition, much of the transplant-specific evidence on prehabilitation and on frailty-score change is derived from observational studies, feasibility work, and small pilots; therefore, causal inference regarding “hard” outcomes (e.g., graft survival or mortality) remains limited.

Generalizability may also be constrained by center-level differences in candidate case-mix, baseline frailty assessment practices, staffing and rehabilitation resources, and variability in listing policies and local health-system factors that influence both frailty measurement and the feasibility of prehabilitation pathways. Finally, several frailty instruments may be susceptible to measurement bias in the presence of baseline disability, language/education barriers, or inter-rater variability; programs should therefore interpret scores within a clinical context and ensure that implementation includes calibration, safeguards against inequitable use, and linkage to standardized optimization pathways.

## Conclusions

Frailty screening can strengthen kidney transplant evaluation by adding objective insight into physiologic reserve, improving shared decision-making, and identifying candidates who may benefit from targeted optimization. A pragmatic model uses rapid screening with selective objective testing, embeds results in multidisciplinary review, and links positive findings to actionable prehabilitation-positioning frailty as a tool for risk stratification and intervention, not exclusion.
